# Lightweight grading method for potato late blight severity based on enhanced YOLOv8-Unet3Plus network

**DOI:** 10.3389/fpls.2025.1616864

**Published:** 2025-09-02

**Authors:** Peisen Yuan, Lushuo Jiang, Zhanghao Cheng, Yixi Tan, Yujia Yang, Cheng He

**Affiliations:** ^1^ College of Artificial Intelligence, Nanjing Agricultural University, Nanjing, China; ^2^ State Key Laboratory of Agricultural and Forestry Biosecurity, College of Plant Protection, Nanjing Agricultural University, Nanjing, China

**Keywords:** AI for science, potato late blight, lightweight model, feature fusion, plant disease phenotyping, deep learning

## Abstract

Artificial intelligence for science is a methodology that integrates artificial intelligence into scientific research to improve the precision and efficiency of data analysis and experimental processes. Specifically in potato late blight severity grading, due to the demand for both accuracy and cost-effective deployment, traditional methods are limited by subjective evaluation and timeconsuming manual measurement. In this paper, a lightweight grading model based on an enhanced YOLOv8-UNet3Plus network is proposed to enable objective and accurate potato late blight severity grading. In detail, the YOLOv8 network is optimized by integrating Spatial and Channel Reconstruction Convolution module, Bi-directional Feature Pyramid Network and Powerful-IoU loss, the UNet3Plus network is optimized by embedding Ghost convolution and Multi-Scale Local Response Attention. Experiments on real-world potato late blight datasets demonstrate that our model achieves an precision of 95.73% for leaf localization and an mean Intersection over Union of 82.65% for infected region segmentation with reduced parameters and computational cost. This AI4Science-based model provides an effective solution for potato late blight severity grading.

## 1 Introduction

In recent years, artificial intelligence for science (AI4Science) has transformed the scientific facility fundamental research and achieved numerous breakthroughs in many frontier fields (Wang et al., 2023; Xu et al., 2021). Through deeply integrating the latest artificial intelligence with scientific methods, this approach advances the analysis and processing of complex scientific data in different fields, such as biological sciences (Bhardwaj et al., 2022), plant disease (Shoaib et al., 2023) et al. Currently, the application of AI4Science has shown remarkable potentials in the field of plant disease phenotype data research, particularly for diseases such as potato late blight (Mohanty et al., 2016; Kamilaris and Prenafeta-Boldú, 2018).

Potato late blight, caused by Phytophthora infestans, is a severe disease that affects global agricultural production, leading to annual yield losses of 20%-30% (Fry et al., 2015). To mitigate these severe agricultural losses and develop resistant varieties (Lu et al., 2021), precise grading of late blight severity is essential for effective disease management and breeding programs (Elumalai and Faritha Banu, 2023). However, conventional manual grading methods are time-consuming and highly dependent on subjective experience, which increases grading costs and leads to inconsistent evaluation results. Compared with manual grading limitations, AI4Science could combine advanced data processing techniques and intelligent analysis methods to improve the accuracy of potato late blight severity grading (Li et al., 2022; Yang et al., 2024a).

As one of the core technologies of AI4Science, deep learning has demonstrated notable advantages in plant disease research with its efficient high-dimensional data processing and automatic feature learning capabilities. Based on these advantages, recent advances in deep learning have brought various powerful models for plant disease research, including HRNet (Wang et al., 2019), ConvNeXt (Liu et al., 2022; Woo et al., 2023; Tang et al., 2023), and ViT (Bhuyan and Singh, 2024; He et al., 2024). These models have achieved high accuracy in the tasks, however, their application in agricultural research is constrained by high computational complexity. Therefore, developing a lightweight intelligent grading method with high accuracy is significant for the analysis of late blight disease of potato (Howard et al., 2019).

To achieve this goal, an accurate and lightweight model for potato late blight severity grading was proposed in this paper. Specifically, by utilizing an improved YOLOv8 network (Miao et al., 2025; Lu et al., 2024; Li et al., 2025) and an enhanced UNet3Plus network (Huang et al., 2020; Chen et al., 2025), our method enabled precise leaf localization and fine-grained segmentation of the infected regions. Then, we combine the localization and segmentation results to evaluate the severity grading for each leaf based on infection area ratios.

**Figure 1:**
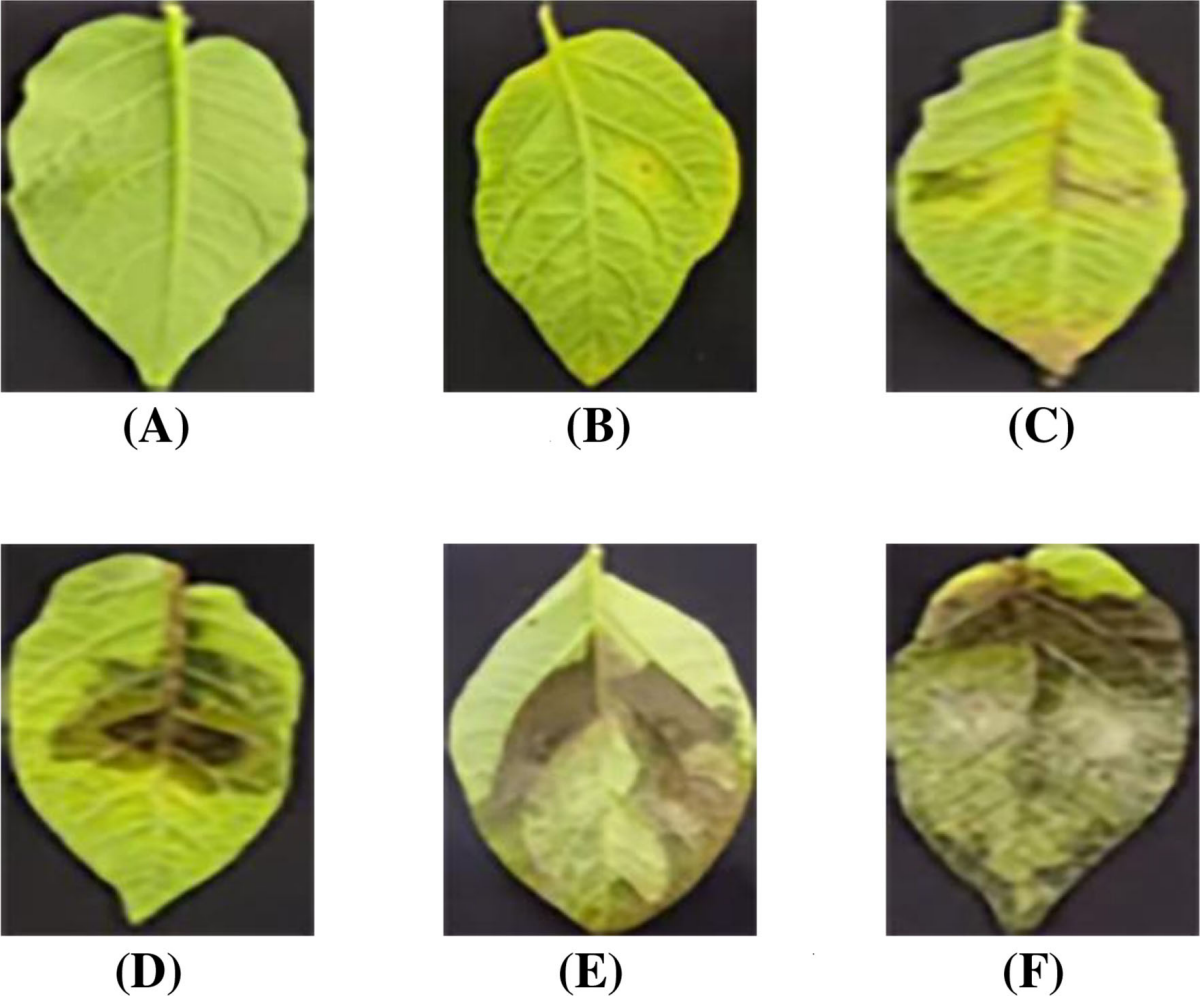
Infection levels of potato late blight. **(A)** Level: 0. **(B)** Level: 1. **(C)** Level: 2. **(D)** Level: 3. **(E)** Level: 4. **(F)** Level: 5.

Besides the development of lightweight and effective grading model, high-quality datasets and scientific evaluation metrics are equally crucial for ensuring reliable potato late blight severity grading. To achieve accurate grading, the inoculation assay with *Phytophthora* infestans was conducted at 6–8 weeks post potato planting (seedling stage with 7–10 compound leaves). Specifically, the 3rd, 4th, and 5th fully expanded compound leaves from the top of each plant were detached. A 40 *µ*L droplet of zoospore suspension (2 × 10^4^ spores/mL) was pipetted onto one side of the mid-vein on the abaxial surface of each leaflet. Three biological replicates were included per material, with each replicate consisting of at least 6 compound leaves. Phenotypic observations and photographic documentation were performed at 5 days post-inoculation (dpi). Based on these images, this paper developed the first open-source potato late blight leaf disease dataset for leaf localization and infected region segmentation, which was further enriched through systematic data augmentation methods. Furthermore, according to infected area proportion, a quantitative grading metric was established to evaluate potato late blight severity. As shown in **Figure 1**, the metrics defines six severity levels (0-5): level 0 (negligible infection, ≤ 0.1%), level 1 (initial symptoms, 0.1% - 10%), levels 2-3 (moderate infection, 10% - 50%), and levels 4-5 (severe infection, *>* 50%). Representative leaf images illustrate the distinct characteristics of each severity level.

The main contributions of this paper are summarized as follows:

1. An open-source dataset of potato late blight leaf disease was constructed for leaf localization and infected region segmentation, providing a reliable foundation for deep learning research in the field of potato late blight disease.
2. An improved YOLOv8-UNet3Plus network is proposed for potato late blight severity grading, which implements accurate and cost-effective disease assessment.
3. A quantitative severity grading system is established based on the proportion of infected leaf area, which provides scientific and objective evaluation metrics for potato late blight grading.

## 2 Methods

### 2.1 System framework

**Figure 2:**
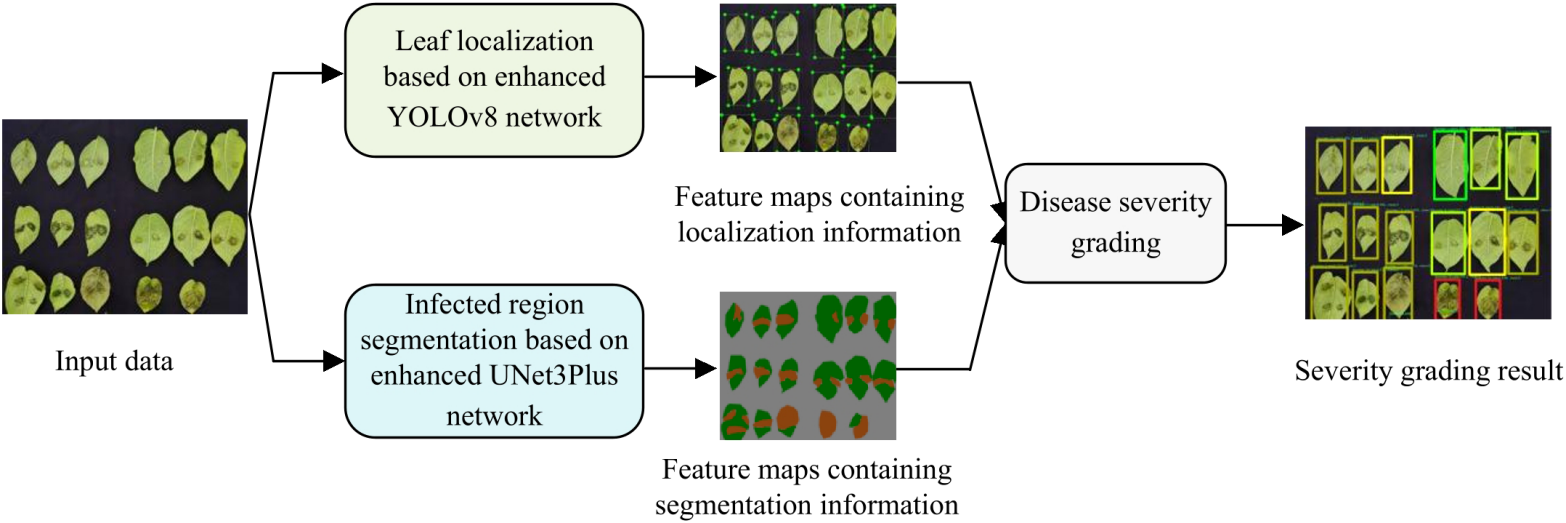
Framework of lightweight potato late blight severity grading.

The proposed model consists of three core processing stages: (1) leaf localization based on enhanced YOLOv8; (2) infected region segmentation based on enhanced UNet3Plus; (3) disease severity grading. Through the integration of these stages, our model effectively implements potato late blight severity grading. Specifically, the processing pipeline shown in **Figure 2** transforms input data through sequential stages to generate severity grading results, which can be summarized as follows:

1. Leaf localization: The enhanced YOLOv8 network processes the input data and generates feature maps containing localization information that enable precise localization of leaves and provide foundational data for downstream analysis.
2. Infected region segmentation: The enhanced UNet3Plus network generates fine-grained segmentation maps that differentiate between healthy and infected regions, providing quantitative data for disease severity assessment.
3. Disease severity grading: In this final stage, the severity level is computed by calculating infected area proportions based on both the leaf localization feature maps and infected region segmentation feature maps, generating quantitative severity grades for each leaf.

### 2.2 YOLOv8-optimized leaf localization

**Figure 3:**
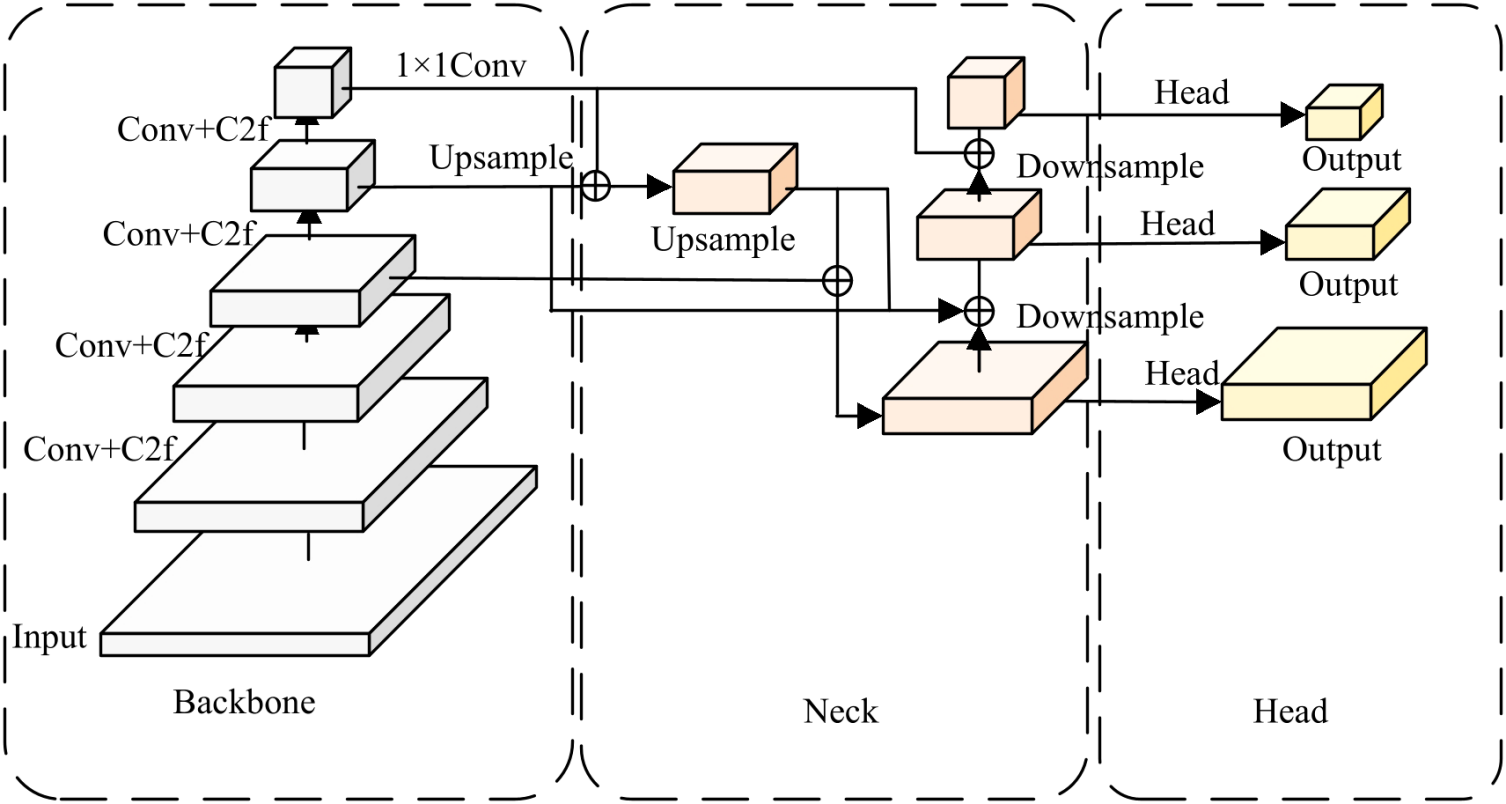
Network structure of leaf location based on YOLOv8.

YOLOv8 is a lightweight single-stage object detection model that implements efficient and accurate leaf localization (Yang et al., 2024b; Orchi et al., 2023; Muthulakshmi et al., 2024). Structurally, **Figure 3** illustrates the architecture of enhanced YOLOv8, which consists of three main components:

1. Backbone: By extracting features from input images through convolutional operations and C2f modules, this component constructs multi-level feature maps to provide fundamental information for the neck network.
2. Neck: The backbone and head networks are connected through this intermediate component, where feature pyramid architecture is adopted for feature fusion and enhancement.
3. Head: Based on features extracted by preceding networks, the head generates final leaf localization results, providing foundational data support for subsequent severity grading.

Based on the baseline YOLOv8n which is the lightest model in the YOLOv8 series, an enhanced network for leaf location is proposed with the following innovations, whose detailed parameters are presented in **Table 1**:

1. ScConv module is integrated into the backbone to implement a lightweight model.
2. BiFPN structure is adopted in the neck to enhance localization capability for leaves which have different sizes.
3. PIoU loss function is adopted to improve the accuracy of leaf bounding box regression.

**Table 1:** Parameters of improved YOLOv8 network.

Network layer	Input size	Output size	*c*
Input	–	640 × 640	3
convolutional layer1	640 × 640	320 × 320	64
convolutional layer2	320 × 320	160 × 160	128
Bottleneck	160 × 160	160 × 160	128
convolutional layer3	160 × 160	80 × 80	256
Bottleneck	80 × 80	80 × 80	256
convolutional layer4	80 × 80	40 × 40	512
Bottleneck	40 × 40	40 × 40	512
convolutional layer5	40 × 40	20 × 20	1024
Bottleneck	20 × 20	20 × 20	1024
SPPF	20 × 20	20 × 20	1024
convolutional layer6	20 × 20	20 × 20	512
upsample	20 × 20	40 × 40	512
BiFPN layer	40 × 40	40 × 40	512
Bottleneck	40 × 40	40 × 40	512
convolutional layer7	40 × 40	40 × 40	256
upsample	40 × 40	80 × 80	256
BiFPN layer	80 × 80	80 × 80	256
Bottleneck	80 × 80	80 × 80	256
convolutional layer8	80 × 80	40 × 40	512
BiFPN layer	40 × 40	40 × 40	512
Bottleneck	40 × 40	40 × 40	512
convolutional layer9	40 × 40	20 × 20	512
BiFPN layer	20 × 20	20 × 20	512
Bottleneck	20 × 20	20 × 20	1024
Detection	[80 × 80,40 × 40,20 × 20]	[80 × 80,40 × 40,20 × 20]	6

#### 2.2.1 ScConv-optimized YOLOv8 backbone network

**Figure 4:**
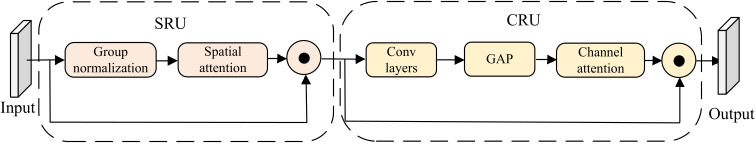
Framework of potato late blight severity grading.

**Figure 5:**
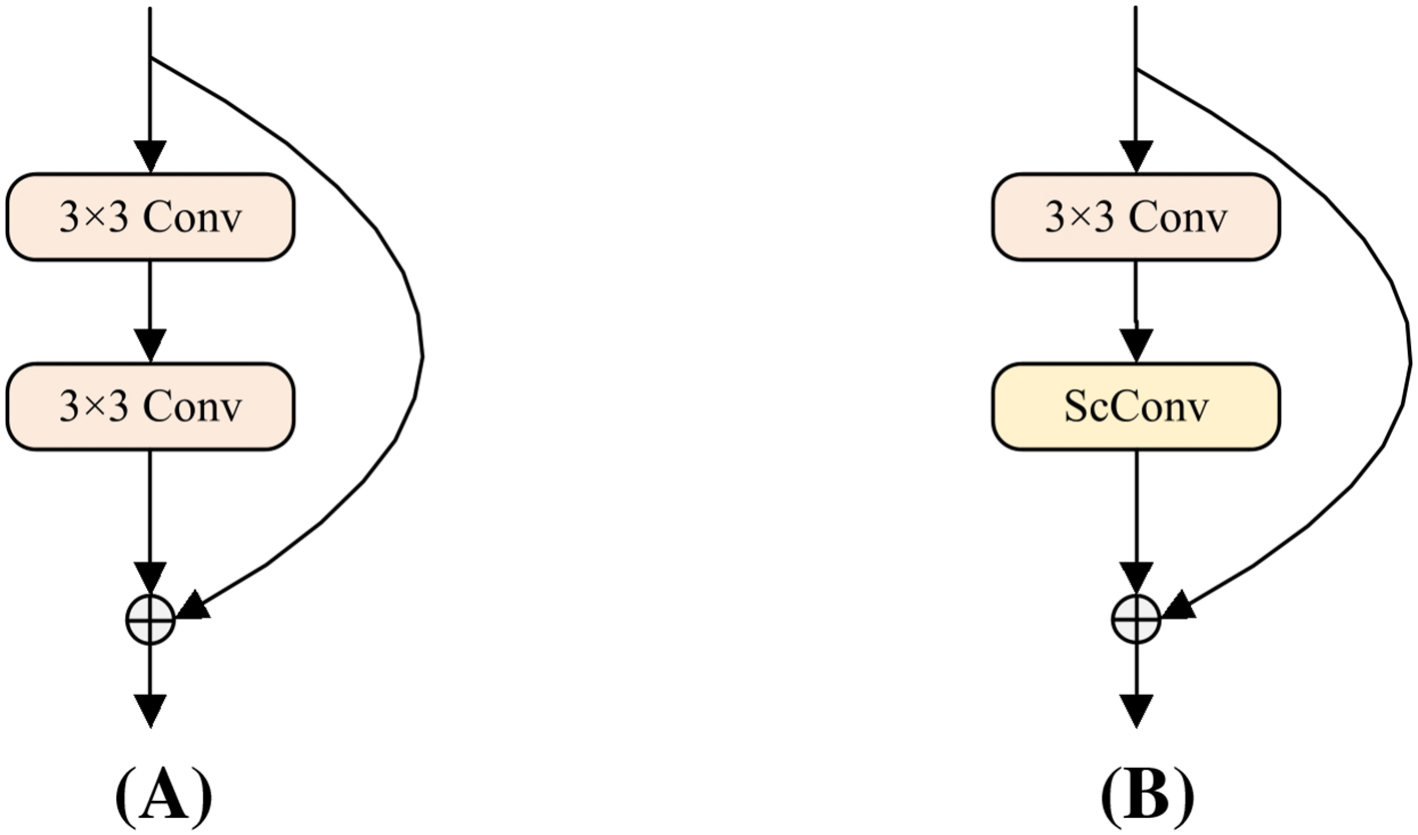
Comparison of YOLOv8 bottleneck and ScConv-Bottleneck.

The ScConv module (Li et al., 2023; Jiang et al., 2024) is a lightweight module that reduces feature redundancy through two units: SRU (Spatial Redundancy Unit) and CRU (Channel Redundancy Unit), whose structure is shown in **Figure 4**. To implement lightweight leaf localization, this paper embeds the ScConv module into the Bottleneck of YOLOv8’s C2f module. In detail, as shown in the comparison between YOLOv8 bottleneck and ScConv-bottleneck in **Figure 5**, ScConv replaces the second 3×3 convolution in the bottleneck, which effectively reduces network parameters. Specifically, the processing pipeline of ScConv module in this paper consists of the following steps:

Step 1: The input feature map is reconstructed along spatial dimensions in the SRU. Given an input feature map $X\in {\mathbb{R}}^{C\times H\times W}$ , SRU first applies group normalization (GN) to the input features, as formulated in Equation 1:


(1) $$\hat{X}g,c,h,w=\gamma_{g}\frac{Xg,c,h,w-\mu_{g}}{\sqrt{\sigma_{g}^{2}+\epsilon }}+\beta_{g}$$

Here, g denotes the group index; $\gamma_{g}$ and $\beta_{g}$ represent learnable affine transformation parameters; $\mu_{g}$ and $\sigma_{g}$ indicate the mean and standard deviation of the *g*-th group; *ϵ* serves as a small constant for numerical stability.

Next, as shown in Equation 2, a spatial attention mechanism is utilized to generate attention map *A_s_* from the normalized features $\hat{X}$ , where *W_s_* is a trainable convolutional kernel, ∗ denotes convolution operation, and *σ*(·) is the sigmoid function.


(2) $$A_{s}=\sigma (W_{s}*\hat{X})$$

After the above processing, the element-wise multiplication (⊙) between attention map *A_s_* and input feature *X* produces the reconstructed feature map *X_s_* as defined in Equation 3:


(3) $$X_{s}=A_{s}⊙X$$

Step 2: Via convolution layers and global average pooling (GAP), *X_s_* is processed through channel-wise refinement to generate a channel attention vector *A_c_* in the CRU. Subsequently, as formulated in Equation 4, the element-wise multiplication between *A_c_* and *X_s_* generates the final output *Y*:


(4) $$Y=A_{c}⊙X_{s}$$

#### 2.2.2 BiFPN-optimized YOLOv8 neck network

**Figure 6:**
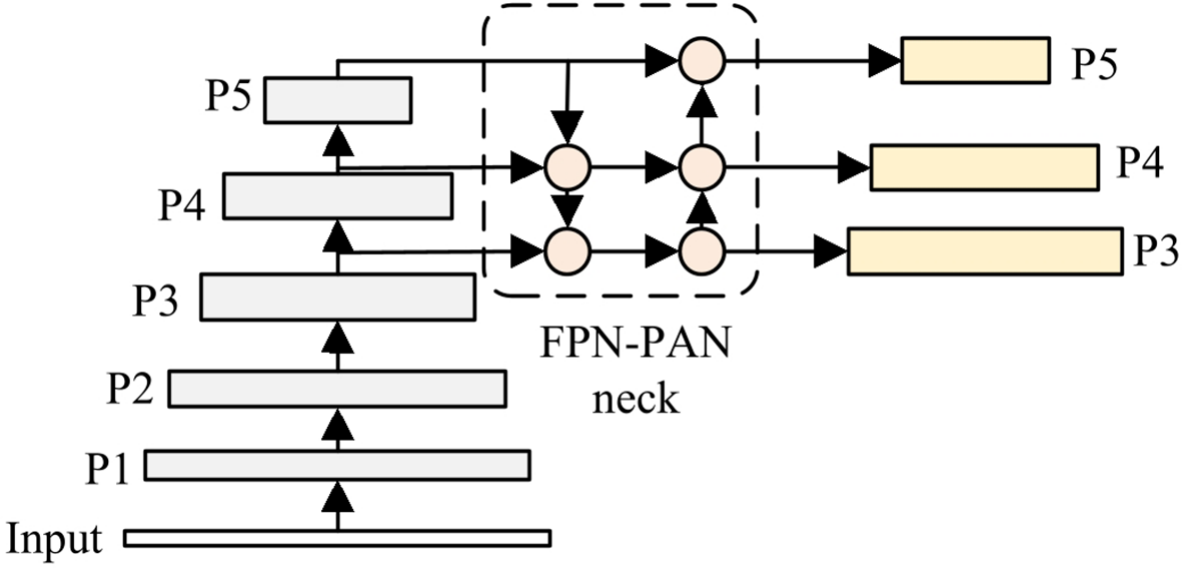
Structure of YOLOv8’s FPN and PAN.

**Figure 7:**
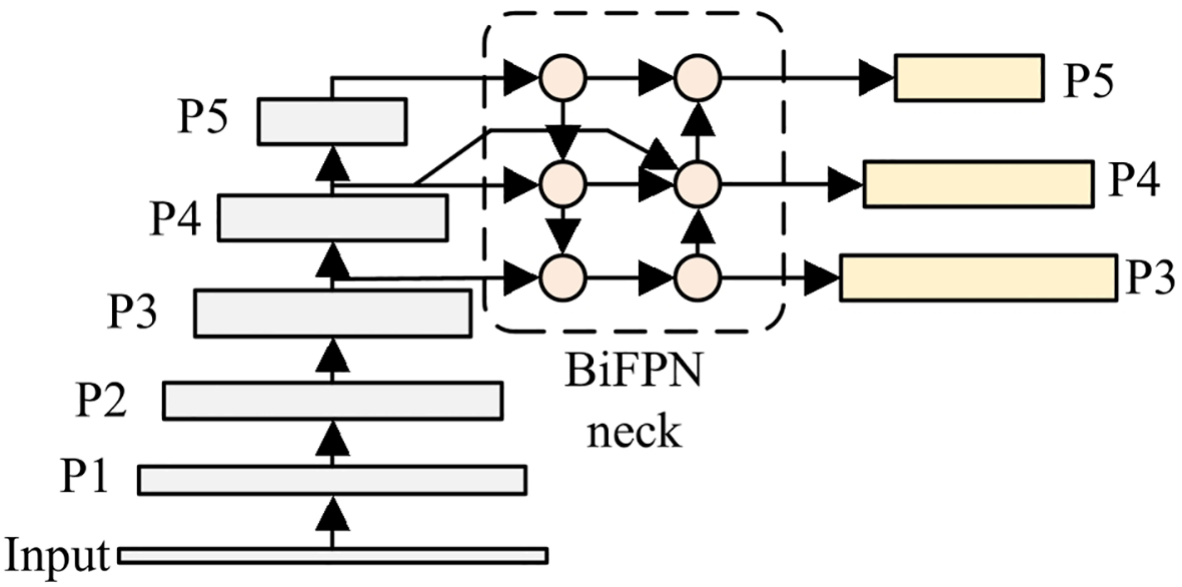
Structure of YOLOv8-BiFPN.

BiFPN (Bidirectional Feature Pyramid Network) is a feature fusion architecture that combines bidirectional paths and skip connections for multi-scale feature processing (Tan et al., 2020; Feng et al., 2025). Through this design, BiFPN enhances feature fusion efficiency and multi-scale object detection capability. Considering these advantageous characteristics, BiFPN is incorporated into the neck of YOLOv8 to handle the wide-ranging scale variations in potato leaves. **Figure 6** shows the FPN and PAN structure which is used in original YOLOv8, and **Figure 7** presents the BiFPN structure. As illustrated, compared to the original FPN and PAN structure, BiFPN introduces additional cross-scale connections and weighted feature fusion, which enables more effective information flow between different scales and enhances the model’s ability to handle multi-scale features.

In reconstructing the neck network, 1 × 1 convolution is initially applied to process the input features, which aims to achieve two objectives: (1) enhance the non-linear representation and semantic expression capabilities; (2) adjust the number of channels, which ensures dimensional compatibility between feature maps and BiFPN input. Subsequent to this initial processing, the features then enter two sequential pathways: top-down and bottom-up.

Top-down pathway: This pathway utilizes upsampling operations to increase feature resolution. At each layer, the upsampled features are combined with backbone features through element-wise addition to merge semantic and spatial information. Let $P_{i}^{\text{td}}\in {\mathbb{R}}^{C_{i}\times H_{i}\times W_{i}}$ denote the output feature map of the top-down pathway at the *i*-th layer, which is computed in Equation 5:


(5) $$P_{i}^{\text{td}}=\text{Conv}(\frac{w_{1}\cdot P_{i}+w_{2}\cdot U(P_{\text{i}+1}^{\text{td}})}{w_{1}+w_{2}+\epsilon })$$

Here, *P_i_* is the backbone feature map; U(·) is the upsampling operation; *w*_1_ and *w*_2_ are learnable weights; *ϵ* is a small constant for stability and Conv(·) is a convolutional layer.

Bottom-up pathway: Features at different scales are fused through downsampling operations in this pathway. Each layer in the bottom-up pathway combines information from three sources: (1) top-down features; (2) previous bottom-up features; (3) backbone features. Let $P_{i}^{\text{bu}}\in {\mathbb{R}}^{C_{i}\times H_{i}\times W_{i}}$ denote the output feature map of the bottom-up pathway at the *i*-th layer, which is evaluated in Equation 6:


(6) $$P_{i}^{\text{bu}}=\text{Conv}(\frac{w_{1}\cdot P_{\text{i}}^{\text{td}}+w_{2}\cdot D(P_{\text{i}-1}^{\text{bu}})+w_{3}\cdot P_{i}}{w_{1}+w_{2}+w_{3}+\epsilon })$$

where D(·) is the downsampling operation, and *w*_3_ is the weight for backbone features.

Finally, these refined features are sent to the head for multi-scale leaf detection.

#### 2.2.3 PIoU-based loss function

In this paper, due to the CIoU loss’s limitation in evaluating bounding box quality for irregularly shaped objects like leaves, the Powerful-IoU(PIoU) loss (Liu et al., 2024) is adopted to replace the CIoU for better leaf location. Compared with CIoU loss, PIoU loss calculates the relative positions between predicted and target boxes’ enclosing matrix, which achieves a more precise balance between box size and position accuracy. Based on this optimized calculation method, PIoU demonstrates particularly effective performance in handling leaves with irregular shapes and varying scales.

Specifically, the PIoU loss function combines the standard IoU loss with a geometric penalty term, as shown in Equation 7:


(7) $$L_{PIoU}=L\text{IoU}+(1-e^{-P^{2}})$$

where LIoU is the intersection-over-union loss between predicted and ground truth boxes, and $1-e^{-P^{2}}$ is the geometry-sensitive penalty component. The penalty coefficient *P* is defined in Equation 8:


(8) $$P=\frac{1}{4}(\frac{dw_{1}}{wgt}+\frac{dw_{2}}{w_{gt}}+\frac{dh_{1}}{h_{gt}}+\frac{dh_{2}}{h_{gt}})$$

**Figure 8:**
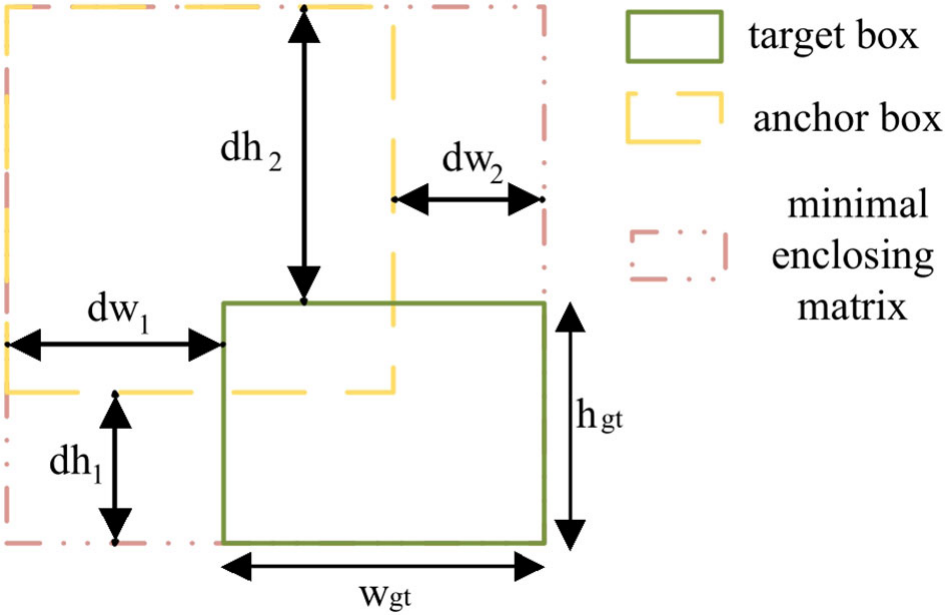
PIoU-based losses.

Here, as shown in **Figure 8**, which illustrates PIoU-based bounding box calculation, $w_{gt}$ and $h_{gt}$ are the width and height of the ground truth box. The width difference terms *dw*_1_ and *dw*_2_ measure the maximum width excess: *dw*_1_ = max(*w_b_*) − *w_gt_* represents the excess between the ground truth box and its minimum enclosing rectangle containing the prediction box, *dw*_2_ = max(*w_p_*) − *w_gt_* represents the excess between the prediction box and their joint minimum enclosing rectangle. The height difference terms *dh*_1_ and *dh*_2_ are defined similarly for the height dimension.

### 2.3 UNet3Plus-optimized infected region segmentation

UNet3Plus is a fine-grained semantic segmentation model that implements pixel-level infected region segmentation. Architecturally, the network consists of two parts:

1. Encoder: The input image sequentially passes through five encoding stages, each of which contains a convolutional layer and a 2 × 2 max pooling layer. Specifically, at each stage, the feature map size is reduced by half, and the number of channels is doubled. Through these encoding operations, a multi-scale feature pyramid is formed.
2. Decoder: The decoder contains five stages. By utilizing feature fusion and convolution operations, each decoder stage integrates features from three sources: (1) the encoder at the same level; (2) upsampled features from other decoder layers; and (3) upsampled features from all deeper encoder stages. Through these decoding operations, pixel-wise infected region segmentation results are generated by the final layer.

**Figure 9:**
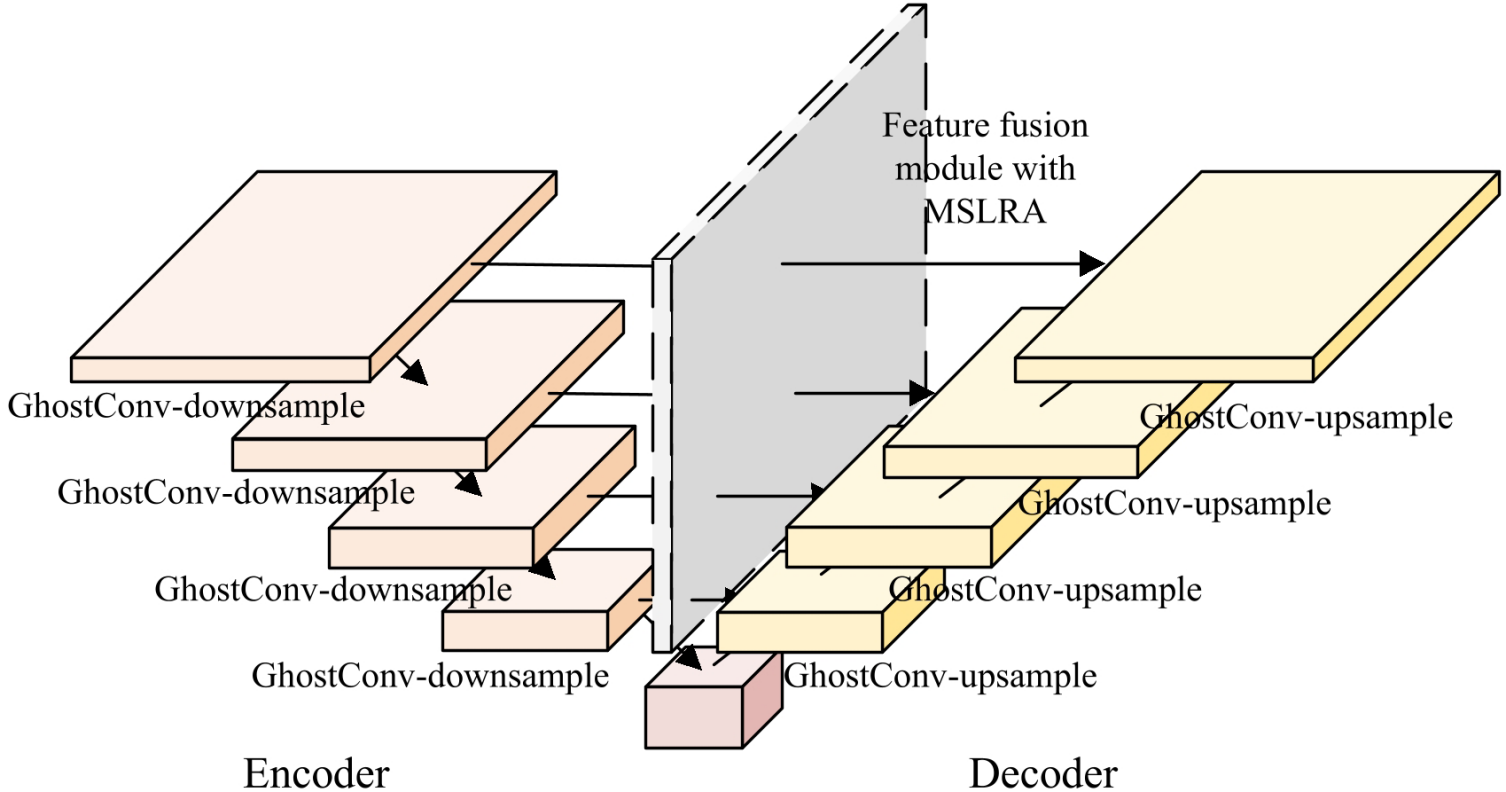
Network structure of infected region segmentation.

Based on the UNet3Plus network, two modifications are implemented in this paper, which can be visualized in the structural diagram presented in **Figure 9**: (1) The convolution modules are replaced with Ghost convolution in both encoder and decoder, which reduces the model parameters; (2) Multi-Scale Local Response Attention(MLSRA) is integrated into the decoder’s feature fusion module to enhance the infected region segmentation performance. **Table 2** presents the network parameters in the infected region segmentation model.

**Table 2:** Parameters of improved UNet3Plus network.

Network layer	Input size	Output size	*c*
Input	–	256 × 256	3
convolutional layer1	256 × 256	256 × 256	64
downsample1	256 × 256	128 × 128	64
convolutional layer2	128 × 128	128 × 128	128
downsample2	128 × 128	64 × 64	128
convolutional layer3	64 × 64	64 × 64	256
downsample3	64 × 64	32 × 32	256
convolutional layer4	32 × 32	32 × 32	512
downsample4	32 × 32	16 × 16	512
convolutional layer5	16 × 16	16 × 16	1024
upsample4	16 × 16	32 × 32	1024
convolutional layer6	32 × 32	32 × 32	64
Feature fusion module	[256 × 256,128 × 128,64 × 64,32 × 32,16 × 16]	32 × 32	320
upsample3	32 × 32	64 × 64	320
convolutional layer7	64 × 64	64 × 64	64
Feature fusion module	[256 × 256,128 × 128,64 × 64,32 × 32,16 × 16]	64 × 64	320
upsample2	64 × 64	128 × 128	320
convolutional layer8	128 × 128	128 × 128	64
Feature fusion module	[256 × 256,128 × 128,64 × 64,32 × 32,16 × 16]	128 × 128	320
upsample1	128 × 128	256 × 256	320
convolutional layer9	256 × 256	256 × 256	64
Feature fusion module	[256 × 256,128 × 128,64 × 64,32 × 32,16 × 16]	256 × 256	320
convolutional layer10	256 × 256	256 × 256	3

#### 2.3.1 Lightweight encoder-decoder module based on Ghost convolution

To implement lightweight model’s construction, Ghost convolution (Tang et al., 2022; Han et al., 2020, 2022) is utilized to replace the conventional convolution operations in both encoder and decoder of UNet3Plus, which reduces the number of parameters and computational cost (Zhang et al., 2024; Bui and Do Le, 2025). Specifically, the Ghost convolution consists of three steps:

Step 1: The Ghost convolution module first extracts enriched feature representations through combining standard and lightweight convolutions, as shown in Equations 9.


(9) F=CheapConv(PrimaryConv(X))⊕PrimaryConv(X)

Here, PrimaryConv(·) and CheapConv(·) represent standard convolution and depthwise separable convolution respectively, and ⊕ denotes feature concatenation.

Then the LightSE module of the Ghost convolution module enhances these concatenated features with cross-dimensional dependencies, as shown in Equations 10:


(10) $$F_{1}=\text{LightSE}(F)$$

Step 2: For efficient feature transformation, depthwise separable convolution is utilized in the Ghost convolution. For input channels *C_in_*, output channels *C_out_*, and kernel size *k*, depthwise separable convolution reduces the parameter count from $O(C_{in}\times C_{out}\times k^{2})$ to $O(C_{in}\times k^{2})$ , which significantly reduces computational complexity.

After the above processing, the Ghost convolution applies channel attention to highlight informative features, as shown in Equations 11 and 12, where *σ* represents the sigmoid function, GAP(·) denotes global average pooling, and ⊙ indicates channel-wise multiplication.


(11) Attn=σ(GAP(F1))

(12) output=X⊙Attn

Similar to the initial step, the subsequent stage of the Ghost module also utilizes both primary and cheap convolutions in parallel to process *output*, as shown in Equations 9, maintaining structural consistency and enhancing feature diversity.

Step 3: By incorporating residual connection, the network enhances feature propagation capability and mitigates information loss, as formulated in Equations 13:


(13) Y=output+Shortcut(X)

#### 2.3.2 Decoder architecture based on MSLRA

In order to improve infected region segmentation accuracy for leaves of different scales and reduce computational complexity, Multi-Scale Local Response Attention (MSLRA) is proposed and integrated into the decoder’s feature fusion module to replace the 1 × 1 convolution. The proposed MSLRA, based on Coord attention (Hou et al., 2021), incorporates a multi-scale local response mechanism that extracts local features through multi-scale windows to generate attention maps, which strengthens the ability to focus on important features at different scales and orientations.

To implement this mechanism, MSLRA processes input features $X\in {\mathbb{R}}^{C\times H\times W}$ through coordinated spatial enhancement and multi-scale enhancement, which effectively captures both global context and local details. In detail, the processing pipeline consists of two main steps:

Step 1: The input features are first compressed by applying global average pooling separately along height and width dimensions, as shown in Equations 14. Here, *z_h_* and *z_w_* preserve the structural patterns.


(14) $$\begin{array}{ll} z_{h}(h) & =\frac{1}{W}\sum_{w=1}^{W}X(h,w)\in {\mathbb{R}}^{H\times 1}\\ z_{w}(w) & =\frac{1}{H}\sum_{h=1}^{H}X(h,w)\in {\mathbb{R}}^{W\times 1} \end{array}$$

Subsequently, these features are concatenated along the channel dimension and processed through a reduction convolution with compression ratio *r* to generate intermediate features $z\in {\mathbb{R}}^{\frac{C}{r}\times (H+W)}$ . Based on these intermediate features, the attention weights are computed as shown in Equation 15, where *W_h_* and *W_w_* denote 1 × 1 convolutional kernels and *σ* represents the sigmoid activation.


(15) $$\begin{array}{ll} a_{h} & =\sigma (W_{h}*z_{h}^{'})\in {\mathbb{R}}^{H\times 1}\\ a_{w} & =\sigma (W_{w}*z_{w}^{'})\in {\mathbb{R}}^{W\times 1} \end{array}$$

Finally, by utilizing element-wise multiplication (⊗) and outer product (⊕) operations between the attention weights and input features, enhanced features *Y* are generated as shown in Equation 16.


(16) $$Y=X⊗(a_{h}⊕a_{w})\in {\mathbb{R}}^{C\times H\times W}$$

Step 2: The enhanced features *Y* from Step 1 are passed through four distinct pathways, each of which consists of two steps. Firstly, each pathway uses adaptive average pooling(*AAP*) to generate feature maps of different sizes: [1 × 1,3 × 3,5 × 5,7 × 7], which enables the model to focus on various scales of information. Subsequently, in each pathway, the number of channels of feature maps is reduced by *Conv*_1×1_ and activated by ReLU activation. Then, the number of channels of the feature maps is restored to its original dimension by *Conv*_1×1_ to expand the feature representation, as shown in Equation 17.


(17) $$att_{i}={\text{Conv}}_{1\times 1}(\text{ReLU}({\text{Conv}}_{1\times 1}(\text{AAP}(Y))))$$

Next, as shown in Equation 18, in order to form a unified feature representation, a learnable weighted mechanism is utilized to fuse the attention maps which are generated from the four pathways. Here, *λ_k_* represents the learnable weight for each pathway.


(18) $$Att=\sum_{k=1}^{4}\lambda_{k}att_{k}$$

Finally, this unified attention map is combined with the original input feature map *Y* to generate the final output, as formulated in Equation 19.


(19) $$\hat{Output}=Y⊗Att$$

### 2.4 Severity grading

In the above two stages, the leaf localization stage generates bounding box *G* for each leaf, and the infected region segmentation stage implements pixel-wise segmentation, categorizing pixels into background pixels, healthy pixels and infected pixels. Subsequently, for each detected leaf, the severity parameter *S* is calculated based on the segmented pixels within its corresponding bounding box *G*, as shown in Equation 20, where *Illness* represents the infected area and *Health* denotes the healthy area.


(20) $$S=\frac{Illness}{Health+Illness}100\%$$

Based on the severity parameter *S*, the infection level *R* of each leaf is graded according to Equation 21:


(21) $$R=\{\begin{array}{c} 0,S\leq 0.001\\ 1,0.001<S\leq 0.1\\ 2,0.1<S\leq 0.25\\ 3,0.25<S\leq 0.50\\ 4,0.50<S\leq 0.67\\ 5,S>0.67 \end{array}$$

## 3 Experimental results

### 3.1 Experimental environment

The hardware environment is 14 vCPU Intel(R) Xeon(R) Platinum 8362 CPU @ 2.80GHz; Memory 45GB; RTX3090 GPU 24GB. The software environment is ubuntu20.04 operating system, Python 3.8, Pytorch 1.10.0, and Cuda 11.3.

### 3.2 Dataset

**Figure 10:**
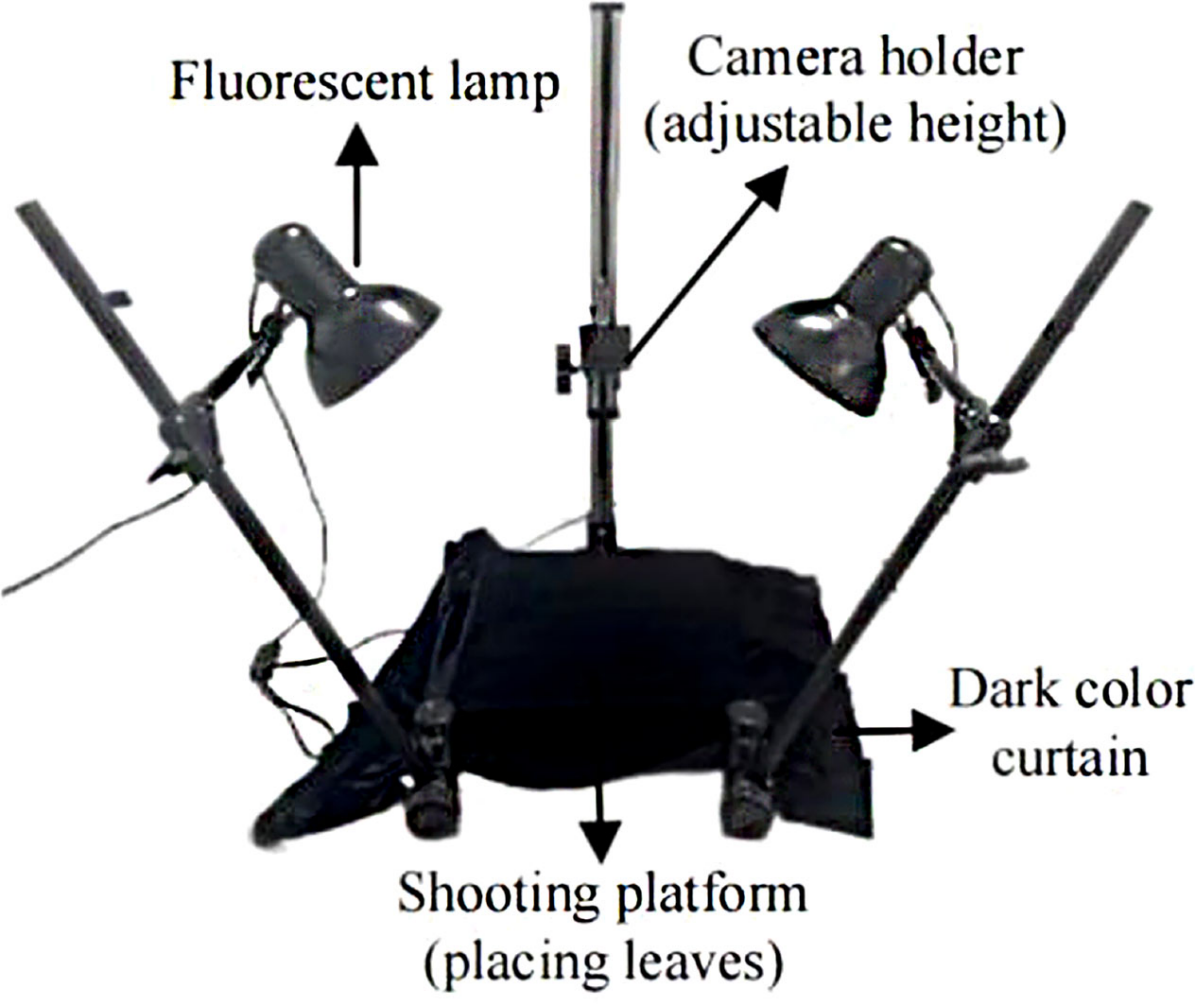
Potato late blight data acquisition equipment.

The dataset consists of approximately 4,700 potato late blight leaf samples across 6 severity levels, which were collected by the equipment shown in **Figure 10**. In this equipment, an adjustable lifting mechanism is featured to capture leaves at different scales, and a flexible lighting system is featured to adapt to varying shooting conditions. With this professional equipment, the collected images are of high quality and show clear gradation in both disease coloration and infection coverage, providing solid support for potato late blight grading.

Furthermore, the data augmentation methods are applied in this paper to enrich the potato late blight dataset. As a fundamental technology in deep learning (Shorten and Khoshgoftaar, 2019), data augmentation enriches the original dataset through various transformation methods (Pandian et al., 2019; Wagle et al., 2021). To meet practical application requirements, different augmentation strategies were adopted for leaf localization and infected region segmentation tasks because of the differing characteristics and challenges of each task.

For leaf localization, we utilized the built-in data augmentation strategy of YOLOv8, which includes standard transformations such as random cropping, scaling, and color adjustments. These augmentations are sufficient for our task because leaf localization primarily requires robustness to variations in leaf position, size, and lighting conditions, which are effectively addressed by YOLOv8’s default augmentations.

For infected region segmentation, more diverse and intensive augmentations were used because of the task’s higher complexity and sensitivity to variations in infection patterns. The following augmentation methods were applied with specific justifications:

1. Horizontal flip(probability 0.5): This augmentation could not only helps the model generalize to different orientations of leaves and infection patterns but also doubles the effective training data by creating mirrored versions of each image.
2. Vertical flip(probability 0.5): This strategy ensures the model learn invariant features regardless of vertical orientation, which is particularly important for capturing spatial symmetry in segmentation tasks.
3. Random 90° rotation(probability 0.5): To enable robust segmentation of irregularly oriented infections which may appear at any angle on leaves, we employed 90° rotation augmentation. This method could not only maintain the pathological feature integrity but also provide essential orientation diversity for model training.
4. Random translation, scaling, and rotation(probability 0.5, rotation angle less than 45°, scale ratio 0.1): By simulating real-world variations in camera distance, angle, and leaf positioning, these augmentations ensure the model handle off-center leaves and improve the generalization ability to field conditions.

These augmentations were chosen to address the specific challenges of infected region segmentation, such as the irregular shapes and sizes of infections, their varying locations on leaves, and the need for precise pixel-level predictions. By introducing controlled variability, the model becomes more robust to the diverse appearances of diseased regions in practical scenarios, ultimately improving generalization to unseen data.

**Figure 11:**
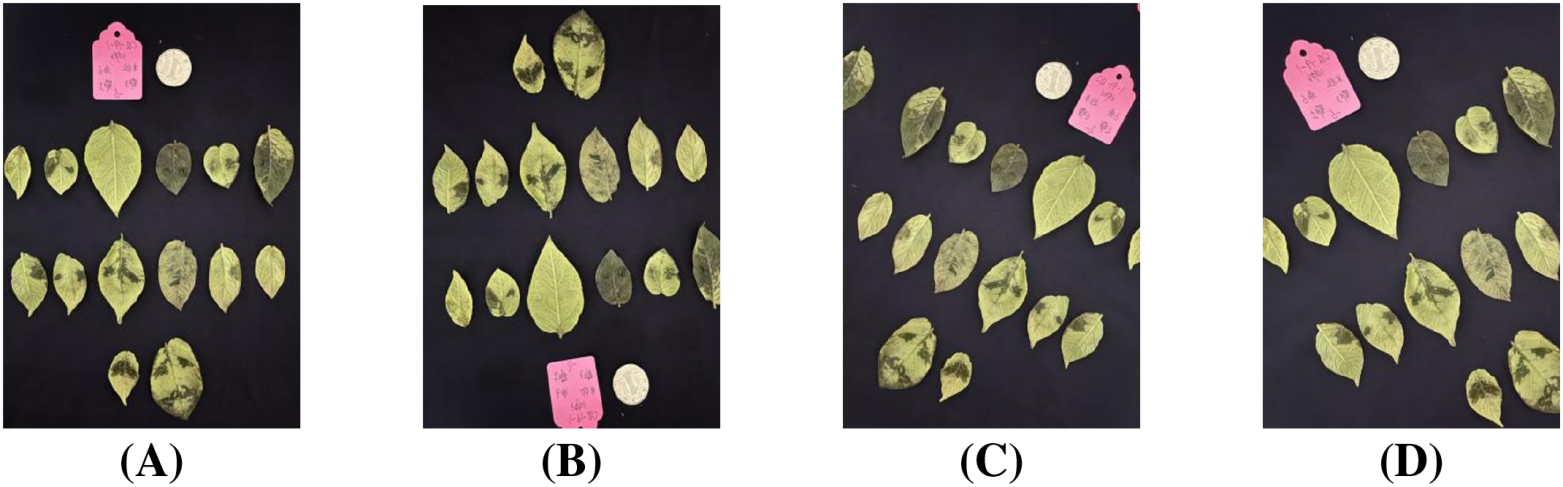
Data augmentation results. **(A)** Original sample. **(B)** Augmented sample. **(C)** Augmented sample. **(D)** Augmented sample.

Through these augmentation methods, the dataset was enriched to approximately 140,000 leaf samples. **Figure 11** demonstrates representative augmentation results of a sample image, illustrating the effectiveness of these transformation methods. What’s more, in order to ensure effective model training and reliable evaluation, the dataset was split into training and testing sets with ratio of 7:3.

### 3.3 Results evaluation

Evaluation metrics provide quantitative measures to assess model performance in deep learning (Sokolova and Lapalme, 2009). Therefore, appropriate metrics selection enables objective comparison among different models and identifies optimal solutions for specific tasks.

#### 3.3.1 Leaf localization

For leaf localization, this paper used Precision, Recall, F1-score and mean Average Precision at 50% Intersection over Union (mAP50) that range between 0 and 1 (Powers, 2011; Ronneberger et al., 2015). In detail, higher values of these metrics indicate better performance. Mathematically, these metrics are defined as Equations 22–25 where *TP_i_* denotes the number of true positive samples, *FP_i_* represents the number of false positive samples, *FN_i_* indicates the number of false negative samples, and *AP*50*_i_* is the Average Precision at IoU threshold 0.5 for class *i*.


(22) $$\text{Precision}=\frac{1}{k}\sum_{i=1}^{k}\frac{TP_{i}}{TP_{i}+FP_{i}}$$

(23) $$\text{Recall}=\frac{1}{k}\sum_{i=1}^{k}\frac{TP_{i}}{TP_{i}+FN_{i}}$$

(24) $$\text{F}1-\text{score}=\frac{2\times \text{Precision}\times \text{Recall}}{\text{Precision}+\text{Recall}}$$

(25) $$\text{mAP}50=\frac{1}{N}\sum_{i=1}^{N}AP50_{i}$$

#### 3.3.2 Infected region segmentation

For infected region segmentation task, precision, recall, F1-score, and mean intersection over union (mIoU) are used to evaluate the model performance (Long et al., 2017). Among these metrics, mIoU is defined as Equation 26:


(26) $$\text{mIoU}=\frac{1}{N}\sum_{i=1}^{N}\frac{TP_{i}}{TP_{i}+FP_{i}+FN_{i}}$$

#### 3.3.3 Model complexity

Model complexity is evaluated through the total number of parameters and floating point operations (FLOPs). In detail, the parameters represent the total trainable weights in the model and FLOPs represent the computational cost of a single forward pass. Both of them indicate a more lightweight and efficient model when their values are lower. Mathematically, these metrics are calculated as Equations 27 and 28, where *L* represents the total number of layers, param*_l_* denotes the number of parameters in layer *l*, and flops*_l_* represents the floating point operations in layer *l*.


(27) $$\text{Parameters}=\sum_{l=1}^{L}{\text{param}}_{l}$$

(28) $$\text{FLOPs}=\sum_{l=1}^{L}{\text{flops}}_{l}$$

### 3.4 Model training and parameters tunning

#### 3.4.1 Training for leaf localization

To achieve high-precision leaf localization, this paper employs the SGD optimizer with a learning rate warmup mechanism to enhance training stability.

**Figure 12:**
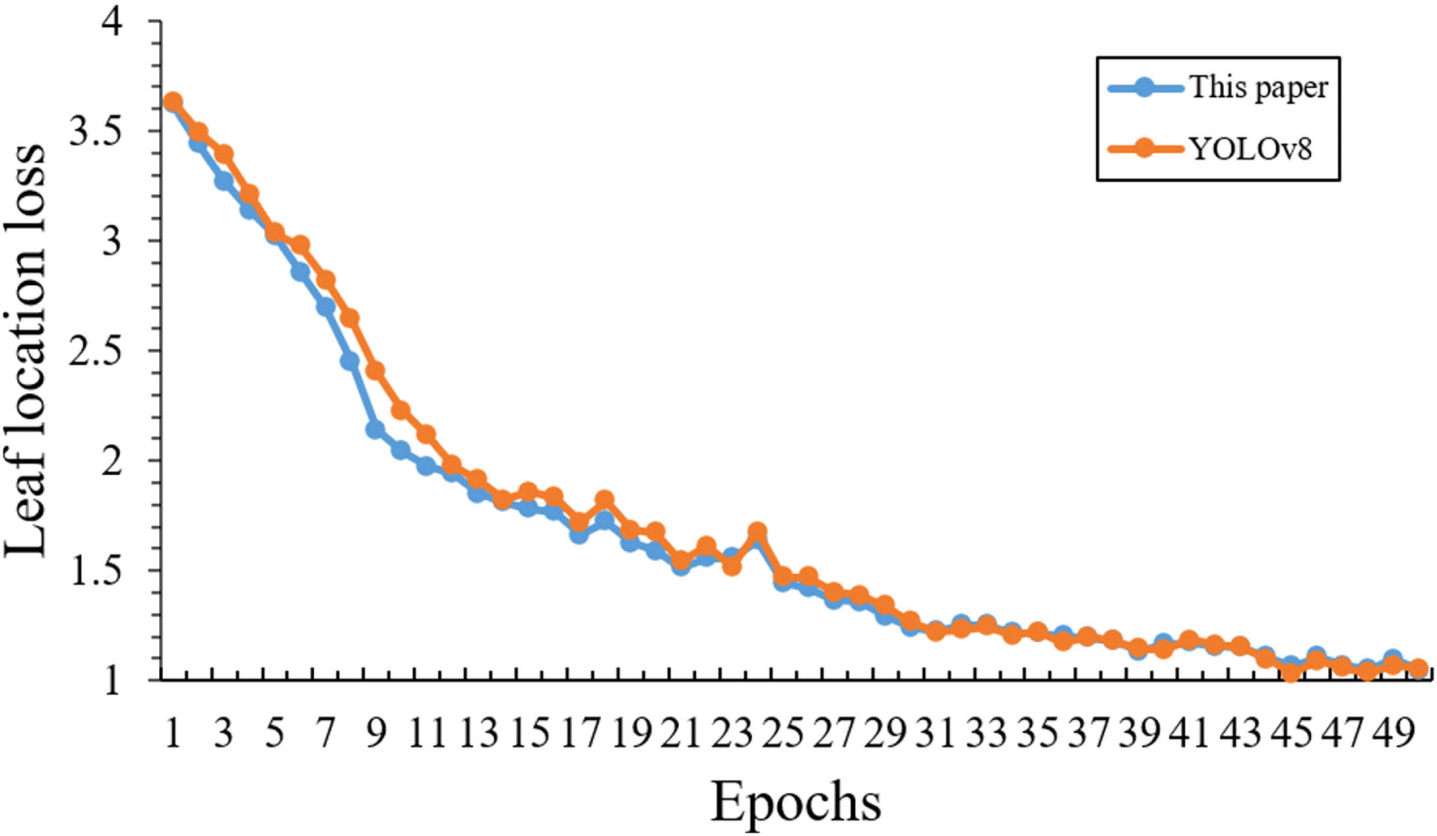
Comparative loss curves of leaf localization.

In order to evaluate the effectiveness of this leaf localization training strategy, this paper analyzed the training process. Specifically, **Figure 12** reveals several distinctive characteristics in train loss curves across different training phases between our enhanced YOLOv8 and the baseline YOLOv8 model, where the horizontal axis represents epochs and the vertical axis represents loss values. Starting from similar initial loss values around 3.62, our model demonstrates superior convergence behavior in multiple phases. Through the first 10 epochs, our model shows a steeper descent trajectory to 2.0433. Then during the intermediate phase (epochs 11-30), our model exhibits a more stable and consistent descent pattern.

Notably, between epochs 20-25, the loss of our model decreases steadily from 1.5894 to 1.4443, suggesting superior learning stability. Finally, in the epochs 31-50, our model achieves a lower loss value than the base model (1.0483 versus 1.0567), which maintains consistent minor oscillations that demonstrate both successful convergence and robust model stability. Based on these training results, it can be concluded that our model achieves better feature extraction capabilities and demonstrates stronger generalization ability.

#### 3.4.2 Training for infected region segmentation

**Figure 13:**
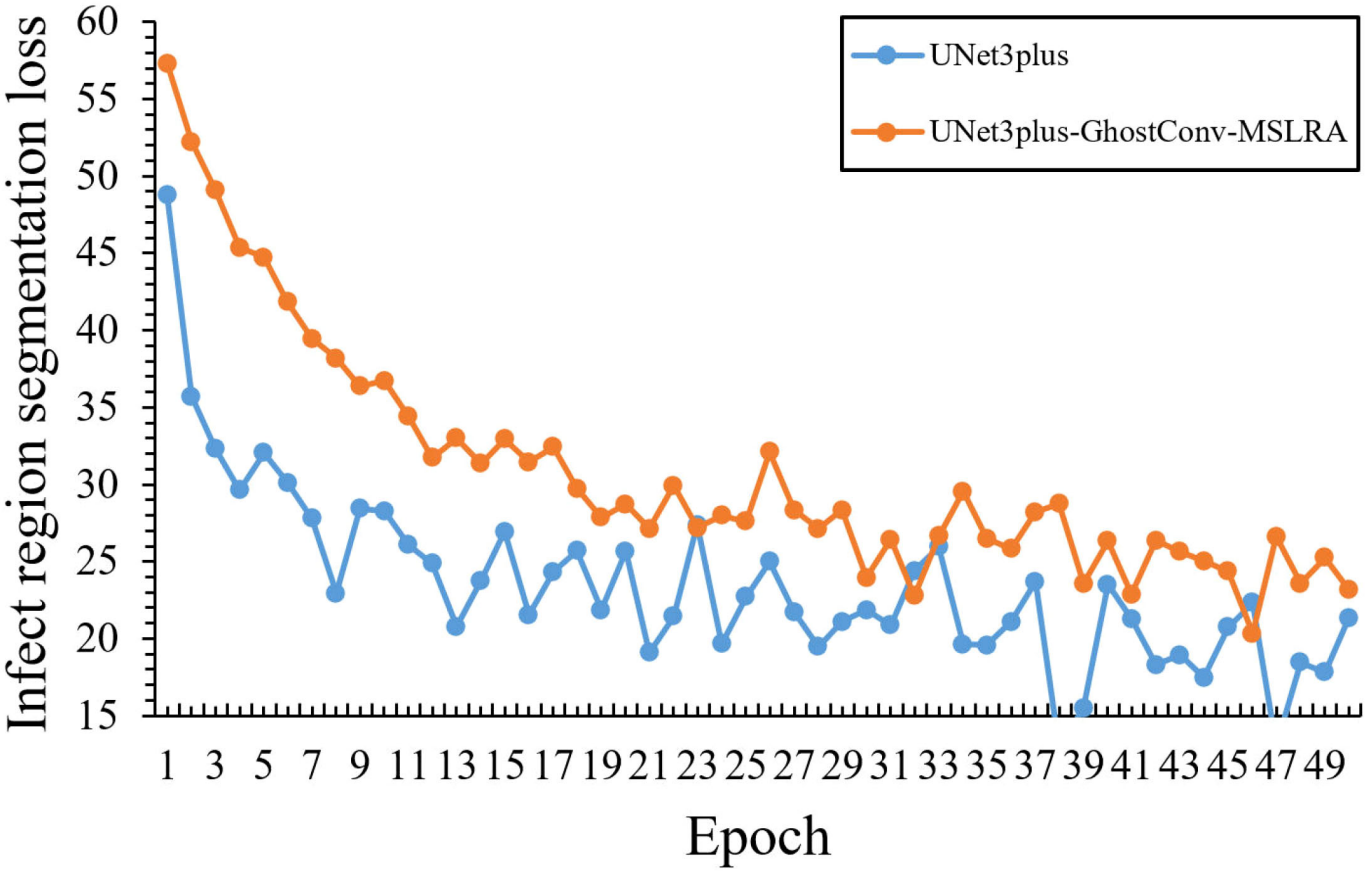
Comparative loss curves of infected region segmentation without optimized training strategy.

In the infected region segmentation task, Adam optimizer (Duchi et al., 2011) combined with WeightDICE loss (Jadon, 2020) function is utilized as the basic training strategy to enable adaptive parameter updates and enhance segmentation accuracy. Initially, **Figure 13** demonstrates the comparative loss curves between our model and the baseline under fixed learning rate, where the horizontal axis represents training epochs and the vertical axis represents loss values. In detail, our model initially showed higher starting loss (57.29) and slower convergence compared to the baseline model (48.75), which was primarily due to our adoption of a lightweight network with fewer parameters that might make it difficult to learn features quickly.

**Figure 14:**
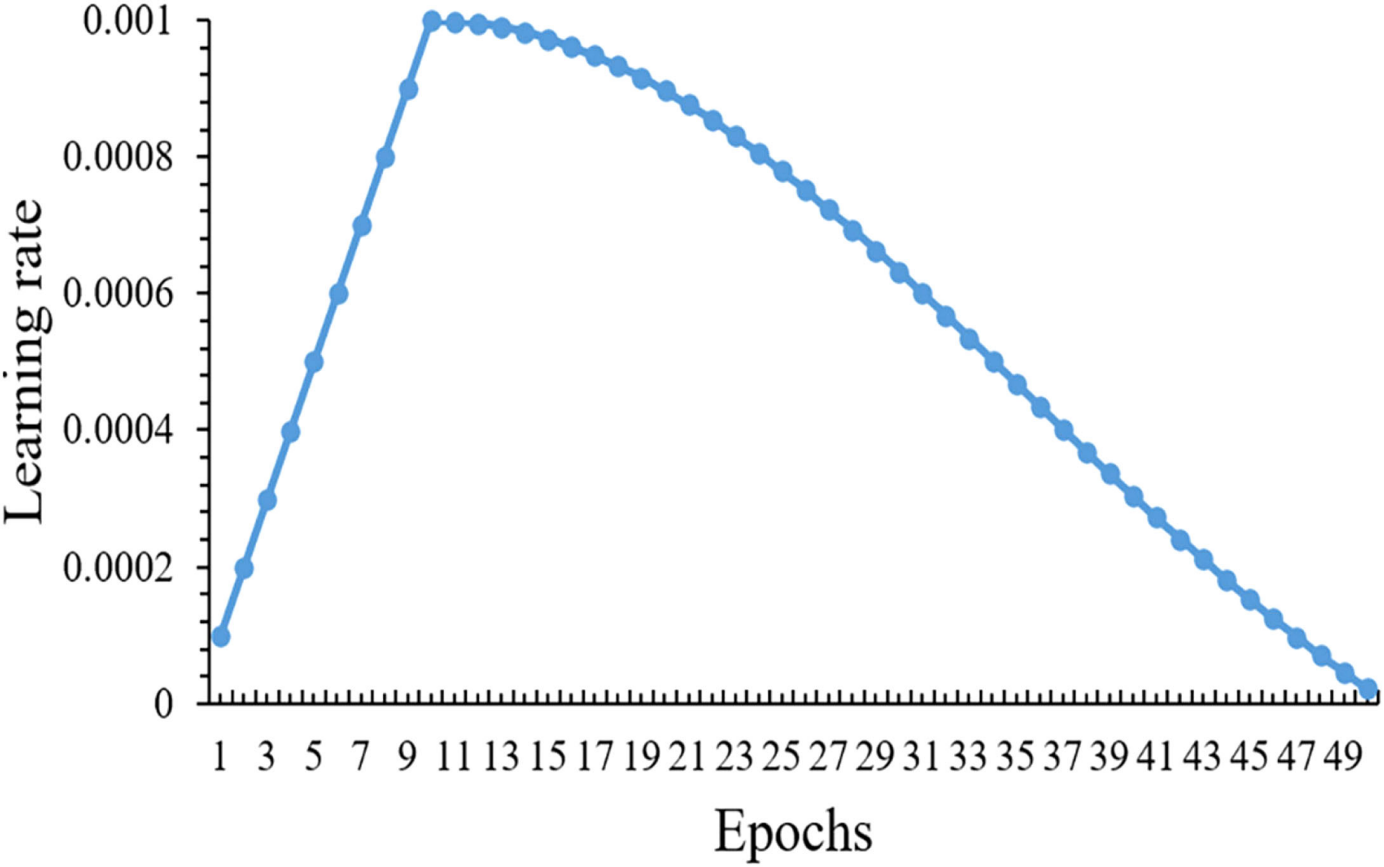
Learning rate schedule under warmup-cosine annealing.

To address the slow convergence issue, an improved learning rate scheduling strategy is designed as shown in **Figure 14**, where the horizontal axis represents training epochs and the vertical axis represents learning rate values. Specifically, the learning rate starts from 0.0001, gradually increases during the first 10 epochs (warm-up phase) to reach 0.001, then follows a cosine curve decreasing to 0.0000225. This strategy was designed considering the characteristics of lightweight models: the warm-up phase allows the model to gradually adapt to data distribution, and cosine annealing enables fine-tuning of parameters in later stages, which maximizes the use of model capacity.

**Figure 15:**
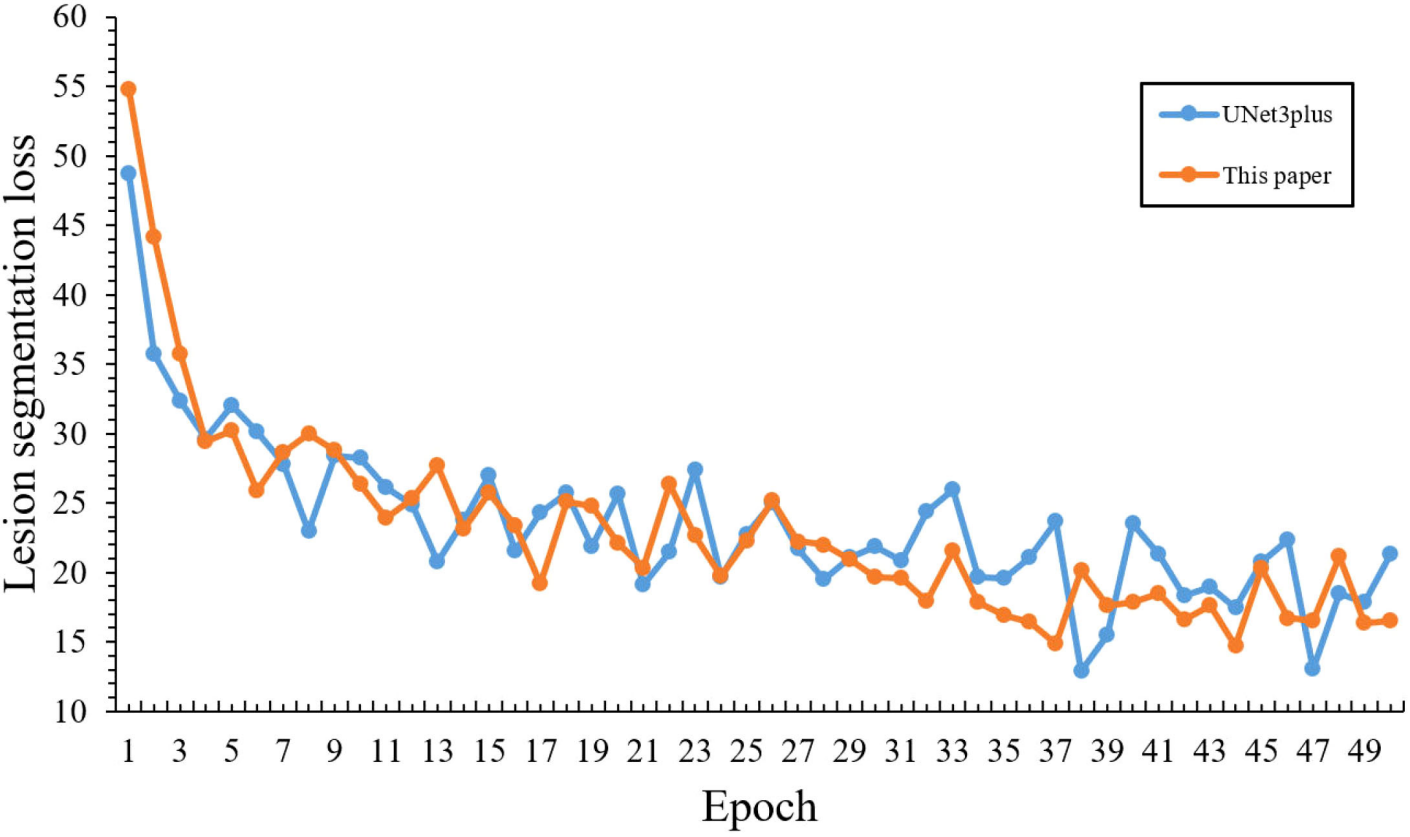
Comparative loss curves of infected region segmentation with optimized training strategy.

Based on the above improved strategy, **Figure 15** demonstrates the comparative training performance between our model and the baseline, where the horizontal axis represents training epochs and the vertical axis represents loss values, revealing that our model effectively overcame the slow convergence challenge of lightweight networks:

Initial training performance: Although our model showed a higher initial loss (54.75) compared to the baseline (48.75) in the first epoch, it quickly achieved better performance by epoch 5 with a loss of 30.18, surpassing the baseline’s 32.05, which demonstrates the effectiveness of our warm-up strategy in overcoming early training challenges.

Training Efficiency: During epochs 10-20, our model’s loss steadily decreased from 26.33 to 22.08, outperforming the baseline model’s range (28.63-25.66). This demonstrates that our learning rate scheduling strategy enabled more efficient parameter optimization during the middle training phase.

Convergence Performance: The most notable improvements emerged during the later training stages (epochs 20-50), where our model’s loss steadily decreased from 22.08 to 16.48. Such stable optimization and superior final performance validate the effectiveness of our learning rate scheduling strategy in addressing the convergence challenges of lightweight models.

### 3.5 Ablation experiments

#### 3.5.1 Ablation experiments of leaf localization

To validate the effectiveness of our proposed components in leaf localization, **Tables 3** and **4** summarize the ablation experiments of this paper, where ‘–’ indicates that the corresponding module is not used in the model and ‘✓’ denotes that the corresponding module is used in the model. According to the results, the baseline YOLOv8 achieves 95.54% precision and 90.89% recall. When integrating ScConv, the model significantly reduces computational complexity, although at the cost of a slight decrease in detection metrics. Subsequently, incorporating BiFPN with ScConv improves the F1 score to 95.94% and maintains computational efficiency, surpassing the baseline performance. Similarly, the integration of PIOU enhances the model’s precision to 96.66%, which demonstrates the highest precision among all configurations. It can be attributed to PIOU’s explicit optimization of localization accuracy. However, this combination shows relatively lower recall compared to our full model, indicating potential over-suppression of positive detections.

**Table 3:** Evaluation results of ablation experiments on leaf localization stage.

ScConv	BiFPN	PIOU	Precision(%)	Recall (%)	F1 (%)	mAP50(%)
–	–	–	95.54	90.89	93.15	93.16
✓	–	–	94.99	88.91	91.84	91.85
✓	✓	–	94.92	89.06	91.89	93.30
✓	–	✓	96.66	90.16	93.29	91.90
✓	✓	✓	95.73	92.78	94.22	94.23

**Table 4:** Parameter and FLOPs comparison of ablation experiments for leaf localization.

ScConv	BiFPN	PIOU	Params(M)	FLOPs(G)
–	–	–	3.01	4.10
✓	–	–	2.82	3.81
✓	✓	–	2.83	3.84
✓	–	✓	2.82	3.81
✓	✓	✓	2.83	3.84

Finally, by combining all three components, our model achieves optimal performance with 95.73% precision, 92.78% recall, and 97.40% F1 score. While the precision of our model is 0.93% lower than the YOLOv8-ScConv-PIOU variant, the difference is offset by two critical advantages:

1. The 2.62% improvement in recall and 0.93% higher F1 score demonstrate our model’s superior overall detection capability.
2. This precision-recall trade-off directly results from BiFPN’s multi-scale feature fusion mechanism, whose intentional preservation of more potential leaf regions across different scales could address the critical agricultural requirement of minimizing missed detections in real scenarios.

Most importantly, our model’s superior performance is achieved with reduced computational complexity (2.83M parameters and 3.84G FLOPs) compared to the baseline model. These experimental results confirm the success of our proposed modifications in both enhancing detection capability and reducing computational costs.

#### 3.5.2 Ablation experiments of infected region segmentation

To evaluate the effectiveness of our proposed components in infected region segmentation, **Tables 5** and **6** summarize the ablation experiments of this paper, where ‘–’ indicates that the corresponding module is not used in the model and ‘✓’ denotes that the corresponding module is used in the model. Firstly, the baseline UNet3Plus achieves a foundation performance with 79.27% precision, 72.35% recall, and 75.65% F1-score. When integrating GhostConv into the network, an improvement in precision could be observed to 81.22% with a slight decrease in recall to 70.49%. Notably, this modification brings significant benefits in computational efficiency, reducing the model complexity from 26.98M parameters and 800.48G FLOPs to 8.45M parameters and 455.84G FLOPs. In a separate experiment, incorporating MSLR demonstrates more substantial improvements in both precision (82.78%) and recall (74.89%), which lead to an enhanced F1-score of 78.64%.

**Table 5:** Ablation experiments metrics for lesion segmentation.

GhostConv	MSLR	Precision(%)	Recall(%)	F1(%)	mIOU(%)
–	–	79.27	72.35	75.65	86.95
✓	–	81.22	70.49	75.47	78.35
–	✓	82.78	74.89	78.64	83.52
✓	✓	85.10	75.44	79.98	82.65

**Table 6:** Parameter and FLOPs comparison of ablation experiments for lesion segmentation.

GhostConv	MSLR	Params(M)	FLOPs(G)
–	–	26.98	800.48
✓	–	8.45	455.84
–	✓	23.41	479.54
✓	✓	4.88	134.40

Building upon these individual improvements, our model, which combines both components, achieves optimal performance with 85.10% precision, 75.44% recall, and 79.98% F1 score. What’s more, this superior performance is achieved with dramatically reduced computational complexity (4.88M parameters and 134.40G FLOPs) compared to the baseline model. These experimental results validate the effectiveness of our proposed modifications in both enhancing segmentation capability and reducing computational costs.

### 3.6 Comparative experiments

#### 3.6.1 Comparative experiments of leaf localization

**Table 7** presents a comprehensive comparison of different models for leaf localization. As shown in the comparison results, our model achieves optimal performance across multiple metrics, demonstrating the highest mAP50 of 97.40% and F1-score of 94.23% with reduced computational resources (3.89G FLOPs). Among the baseline models, YOLOv11 achieves the second-best performance with 96.56% mAP50 and 93.92% F1-score, followed by YOLOv8 with 95.75% mAP50 and 93.16% F1-score. What’s more, YOLOv10 exhibits the lowest performance across all metrics, with both precision and recall around 72%. Additionally, despite achieving the highest precision of 99.78%, FCOS (Tian et al., 2019; Yehia et al., 2024) shows significantly lower recall (74.09%), resulting in a reduced F1-score of 85.04%. Moreover, FCOS requires substantially higher computational resources with 32.12M parameters, approximately 11.37 times larger than our model’s 2.83M parameters. Notably, DETR achieves near-perfect precision of 99.64% but limited recall of 79.28%, resulting in an intermediate F1-score of 88.30%. This performance comes at the cost of heavy computational overhead, which makes it impractical for field deployment.

**Table 7:** Evaluation metrics for leaf localization stage.

Model	Params(M)	FLOPs(G)	Precision(%)	Recall(%)	F1(%)	mAP50(%)
FCOS	32.16	19.88	99.78	74.09	85.04	83.26
DETR	41.28	10.27	99.64	79.28	88.30	86.79
YOLOv8	3.01	4.10	95.54	90.89	93.16	95.75
YOLOv10	2.71	4.20	72.43	72.68	72.56	78.65
YOLOv11	2.62	4.34	96.67	91.31	93.92	96.56
This paper	2.83	3.89	95.73	92.78	94.23	97.40

From the above experiments, we found that there exists a consistent precision-recall trade-off in highparameter models. Both FCOS and DETR sacrifice 20%-25% recall for less than 4%-5% precision improvement, which is a suboptimal balance for agricultural applications that prioritize comprehensive detection over individual prediction confidence. This phenomenon primarily stems from the fundamental architectural biases of FCOS detector and Transformer-based DETR, which intrinsically prioritize highconfidence predictions at the expense of diminished sensitivity to small targets. Therefore, their inherent limitations in cross-scale leaf detection sensitivity consequently result in 15%-20% lower recall rates compared to YOLO-series models.

#### 3.6.2 Comparative experiments of infected region segmentation

**Table 8** presents a comprehensive comparison of different models for infected region segmentation. As demonstrated in the comparison results, our model achieves optimal performance with the highest precision (85.10%), recall (75.44%), and F1-score (79.98%), and requires significantly lower computational resources (134.90G FLOPs and 4.88M parameters). Among the baseline models, UNet3Plus demonstrates the highest mIOU of 86.95% and the second-best F1-score of 75.65%, but requires substantially higher computational cost (800.48G FLOPs), approximately 5.9 times larger than our model. UNet and SegNet (Badrinarayanan et al., 2017) exhibit moderate performance with F1-scores of 72.03% and 74.76% respectively, and PSPNet (Zhao et al., 2017) shows the lowest performance with an F1-score of 59.29%.

**Table 8:** Evaluation metrics for infected region segmentation stage.

Model	Params(M)	FLOPs(G)	Precision(%)	Recall(%)	F1(%)	mIOU(%)
UNet	39.39	322.21	76.18	68.31	72.03	84.01
SegNet	29.44	160.83	82.75	68.18	74.76	75.99
PSPNet	525.50	201.98	67.96	52.58	59.29	67.41
UNet3Plus	26.98	800.48	79.27	72.35	75.65	86.95
This paper	4.88	134.90	85.10	75.44	79.98	82.65

#### 3.6.3 Comparative experiments of attention mechanism

**Table 9** presents a comprehensive comparison of different attention mechanisms under identical experimental conditions. By using the same Ghost convolution-based UNet3Plus architecture, all models were evaluated with consistent hyperparameters, which includes learning rate, training epochs and optimizer settings. In addition, each attention mechanism was integrated at the same position in the network architecture to ensure fair comparison.

**Table 9:** Comparative experiments on attention mechanisms.

Model	Precision(%)	Recall(%)	F1(%)	mIOU(%)
SE	82.76	70.33	76.07	77.94
ECA	83.48	68.73	75.37	78.25
CoordA	83.93	73.56	78.40	80.84
This paper	85.10	75.44	79.98	82.65

As shown in the comparison results, our model achieves optimal performance across multiple metrics, with the highest precision (85.10%), recall (75.44%), and F1-score (79.98%). Among the baseline mechanisms, CoordA demonstrates the second-best performance with a precision of 83.93%, recall of 73.56%, and F1-score of 78.40%. For the other baseline mechanisms, SE (Hu et al., 2018) achieves an F1-score of 76.07% (precision 82.76%, recall 70.33%), and ECA (Wang et al., 2020) yields an F1-score of 75.37% (precision 83.48%, recall 68.73%).

### 3.7 Results visualization

#### 3.7.1 Results visualization of leaf localization

**Figure 16:**
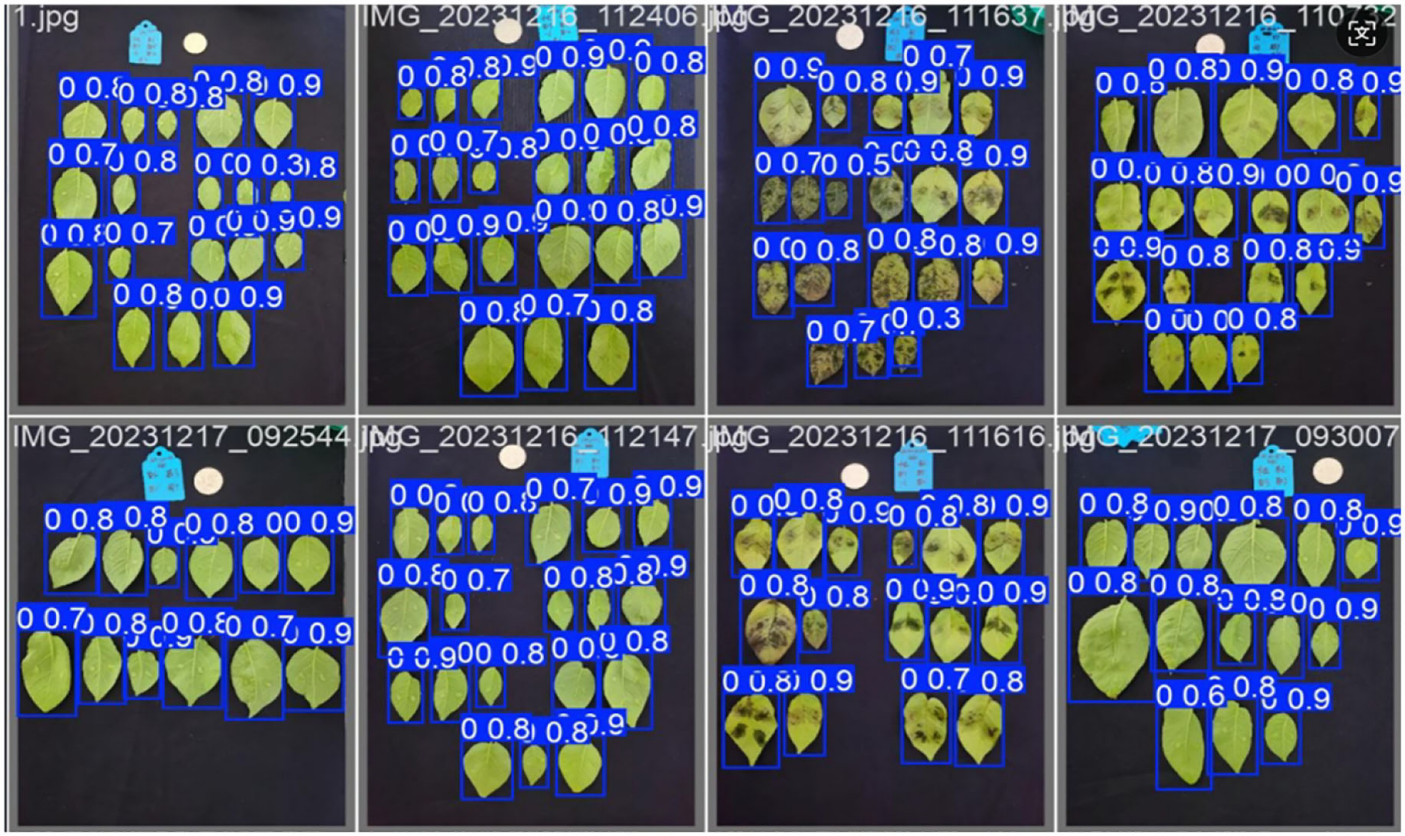
Visualization of leaf localization results.

**Figure 16** illustrates representative leaf localization results from test set samples. Specifically, the generated bounding boxes precisely encompass the target leaves and minimize background inclusion, which effectively handles variations in leaf size, shape, and orientation. Through these visualization results, our model’s robust leaf localization capability has been validated.

#### 3.7.2 Results visualization of infected region segmentation

**Figure 17:**
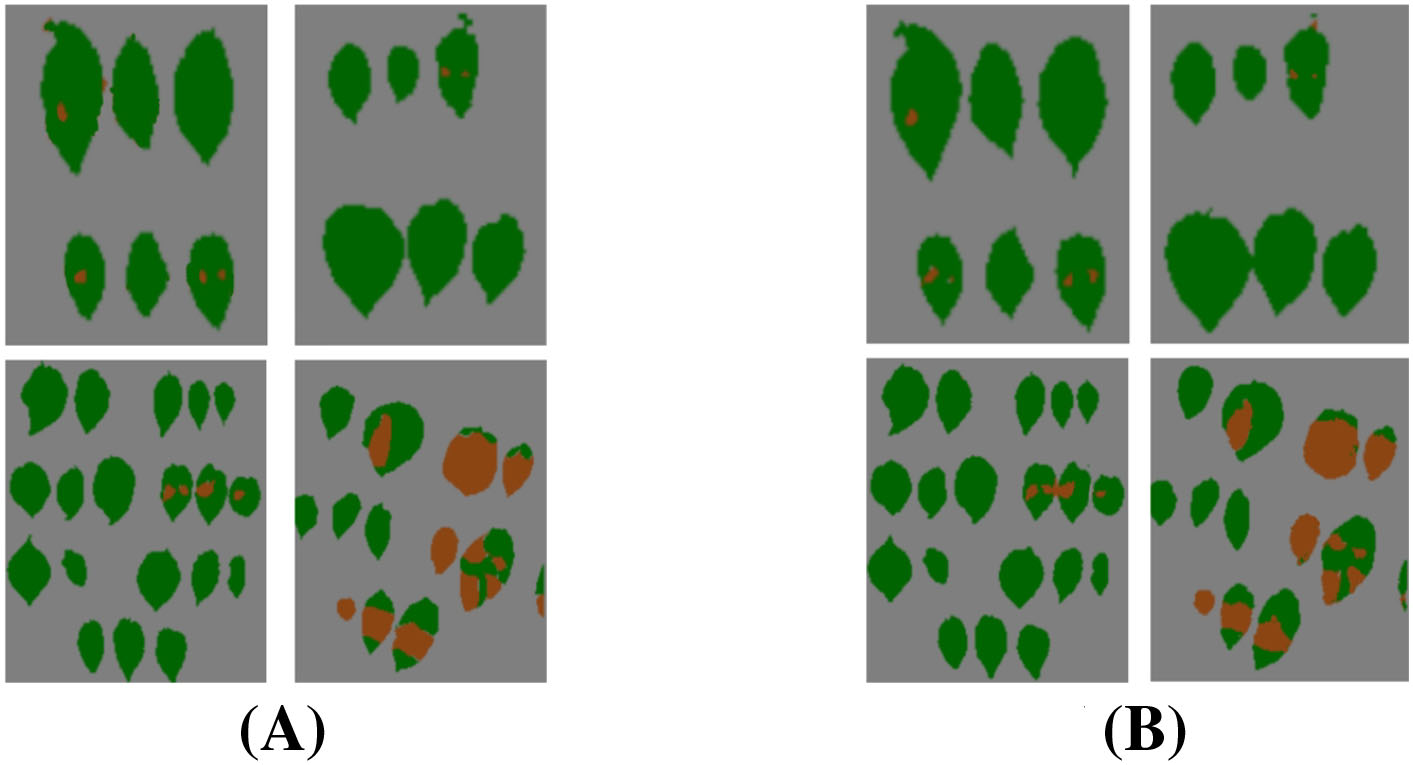
Visualization of infected region segmentation results. **(A)** Segmentation labels. **(B)** Predicted results.

**Figure 17** demonstrates that the segmentation results achieve high consistency with ground truth annotations. Based on these visualization results, our model exhibits three significant advantages: (1) accurate boundary delineation of irregular infected regions, particularly in areas with complex morphological characteristics; (2) reliable segmentation of small infection spots, even for early-stage lesions with subtle features; (3) effective suppression of false positives in healthy tissue areas, maintaining high specificity in disease identification. These results comprehensively demonstrate our model’s superior capability in fine-grained infected region segmentation.

#### 3.7.3 Results visualization of severity grading

**Figure 18:**
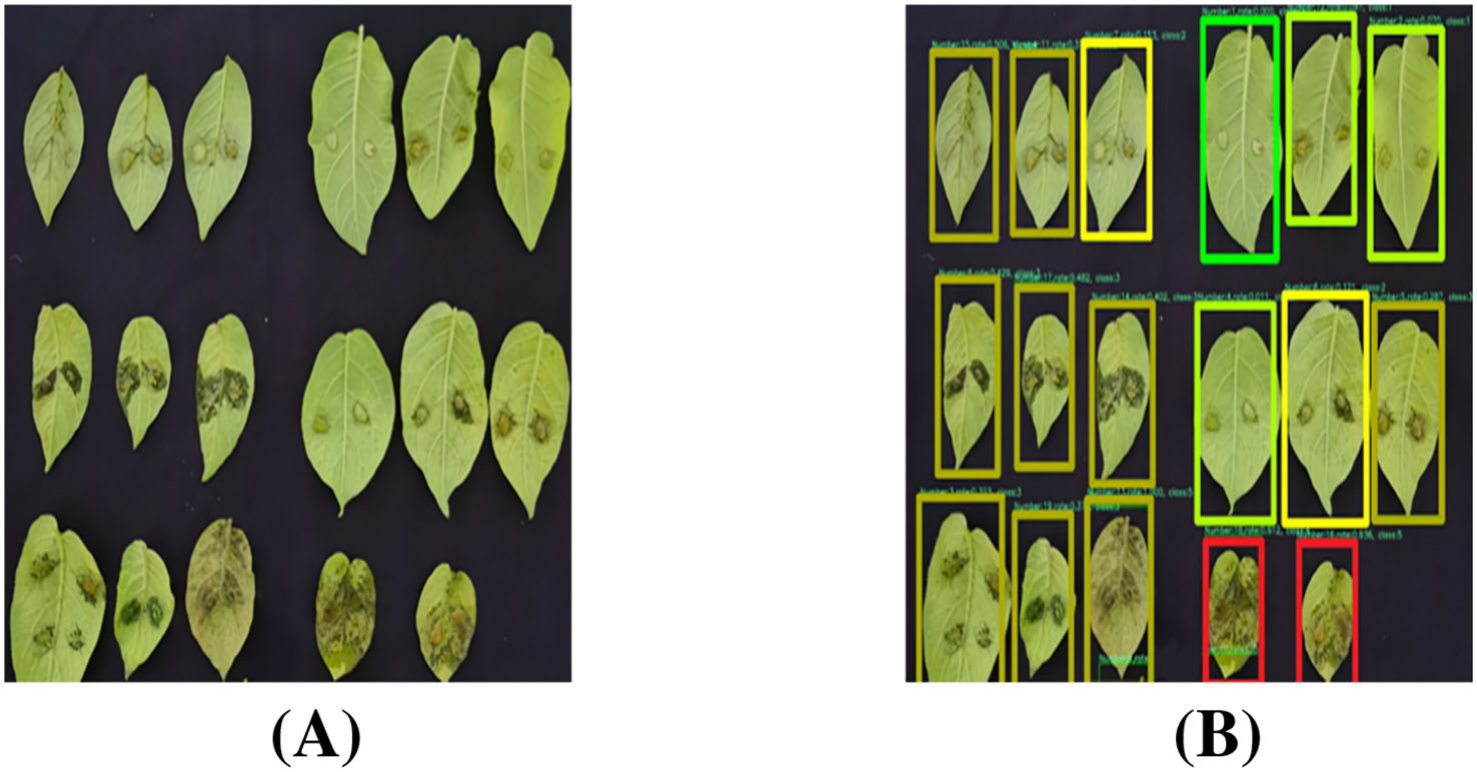
Visualization of severity grading results. **(A)** Ungraded data. **(B)** Grading results.

**Figure 18** illustrates the practical application of our potato late blight severity grading model. Validated by the consistent grading results, the area-based grading method demonstrates two principal advantages: (1) scientific and accurate severity grading through precise quantification of infected regions, and (2) efficient batch processing capability for applications.

## 4 Conclusions

1. Dataset for potato late blight leaf disease is collected in this paper, focusing on leaf localization and infected region segmentation tasks. This dataset establishes a reliable foundation for deep learning research in potato late blight disease grading.
2. The severity grading metric based on infected leaf area proportion is established, which transforms traditional experience-based assessment into standardized evaluation. This metric enables objective evaluation of potato late blight severity through precise calculation of infection ratios.
3. A lightweight deep learning model utilizing enhanced YOLOv8-UNet3Plus network is proposed to enable accurate potato late blight severity grading. In terms of individual components, the enhanced YOLOv8 achieves superior leaf localization performance with an F1-score of 94.23% and mAP of 97.40% and an 5.86% reduction in parameters. Similarly, the enhanced UNet3Plus demonstrates improved infected region segmentation with an accuracy of 82.65% and an 87.17% reduction in parameters. Through these optimization, our model demonstrates exceptional efficiency and accuracy for potato late blight severity grading.

## Data Availability

The data analyzed in this study is subject to the following licenses/restrictions: be requested. Requests to access these datasets should be directed to peiseny@163.com.
